# The Vertebral Bone Marrow clot as cell therapy and multifunctional bioscaffold for spinal fusion surgery: protocol for a randomized clinical trial

**DOI:** 10.3389/fmed.2025.1591041

**Published:** 2025-05-09

**Authors:** Maria Sartori, Giuseppe Tedesco, Paolo Spinnato, Antonio Mazzotti, Alessandro Gasbarrini, Cesare Faldini, Marco Miceli, Gianluca Giavaresi, Francesca Salamanna

**Affiliations:** ^1^Surgical Sciences and Technologies, IRCCS Istituto Ortopedico Rizzoli, Bologna, Italy; ^2^Department of Spine Surgery, IRCCS Istituto Ortopedico Rizzoli, Bologna, Italy; ^3^Diagnostic and Interventional Radiology, IRCCS Istituto Ortopedico Rizzoli, Bologna, Italy; ^4^1st Orthopaedic and Traumatologic Clinic, IRCCS Istituto Ortopedico Rizzoli, Bologna, Italy; ^5^Department of Biomedical and Neuromotor Science-DIBINEM, University of Bologna, Bologna, Italy

**Keywords:** Vertebral Bone Marrow clot, cell therapy, multifunctional bioscaffold, spinal fusion surgery, protocol, randomized clinical trial

## Abstract

**Introduction:**

Spinal fusion (SF) represents a commonly performed orthopedic surgical procedure, and to enhance its success rate, various adjunctive therapies have been explored, including the application of local autografts, bone allografts, and different formulations of Bone Marrow Aspirate (BMA). Among these, Vertebral Bone Marrow Aspirate (vBMA) clot emerges as an innovative and promising avenue for future development. The primary challenge in using vBMA clot for SF procedures lies in the lack of high-level clinical evidence demonstrating whether its combination with bone allograft chips is superior to using bone allograft chips alone. Additionally, there is a shortage of preclinical studies investigating the intrinsic biological, anti-inflammatory, and antibacterial properties of vBMA clots.

**Methods and analysis:**

Patients with degenerative lumbar spine diseases (aged 18–80 years, with ≤5 levels involved) are randomly assigned in a 1:1 ratio to undergo SF with autologous vBMA clot associated with bone allograft chips or bone allograft chips alone by a blinded experimenter, who is not involved in the RCT. The primary aim is to evaluate the SF rate and time in the two groups as well as consider age and gender differences. SF rate and time are evaluated with CT scans and x-rays and performed preoperatively and at 1, 3, 6, and 12 months of follow-up (FU). Using the Brantigan classification, quantitative CT analyses of facet joint space density are calculated at baseline and in follow-up controls to complement the standard qualitative visual scale. CT and X-rays will be assessed in a blinded manner by a diagnostic and interventional radiologist. The secondary outcome is to evaluate the pseudoarthrosis rate estimated using the Brantigan classification, and the clinical outcomes are assessed using the Visual Analog Scale (VAS), the Oswestry Disability Index (ODI), and the Short Form 36 Health Survey (SF-36) questionnaire by comparing baseline (pre-surgery) values with those at 1, 3, 6, and 12 months of FUs. All scores and the questionnaire will be evaluated by a blinded assessor. Complications are also recorded and classified as early (major and minor) or late, according to the time of occurrence and findings at 12 months post-surgery. Finally, the preclinical project outcome is to define the biological and anti-inflammatory properties of vBMA clot and demonstrate its antibacterial effect, assessing age and gender differences and including an examination of their interactions.

**Clinical trial registration:**

ClinicalTrials.gov, identifier NCT05947175.

## Introduction

The use of spinal fusion (SF) procedures has significantly increased over the past decade to stabilize the spine in degenerative, oncologic, and traumatic conditions (1). Despite advancements in spinal surgery, pseudarthrosis remains a concern, occurring in approximately 25–35% of procedures (2–6). Several factors influence fusion rates, including patient characteristics (e.g., age and osteoporosis) and treatment-related aspects (e.g., number of levels treated, use of instrumentation, interbody grafts, and surgical approach) (7). With the increase in aging population and the growing need for multilevel spinal fusion surgery, there is a demand for techniques that enhance SF success rates and reduce pseudarthrosis (8). While iliac crest autograft and local autograft (spinous processes/laminae) are considered the gold standard, their use is limited by supply constraints and associated complications (1, 9). Bone allografts serve as substitutes for autografts and can be combined with Vertebral Bone Marrow Aspirate (vBMA) instead of iliac crest bone marrow aspirate to enhance osteogenic properties (10–12). vBMA is readily accessible in spinal surgery during the preparation for pedicle screw insertion and contains ⁓70% more osteoprogenitor cells—derived from mesenchymal stem cells (MSCs)—than iliac crest aspirates (13, 14). Studies have also shown that MSCs from vBMA exhibit high expression levels of Bone Gamma-Carboxyglutamate Protein (BGLAP), Alkaline Phosphatase (ALPL), and Runt-Related Transcription Factor 2 (RUNX2) genes, which are key regulators of the mineralization process, along with specific Hox gene expression, which influences vertebral morphology (13–15). Furthermore, despite vertebrae being sites of high bone turnover and among the first affected by age-related osteoporosis, the biological characteristics of vBMA remain unaffected by donor age (16, 17). However, the primary limitations of vBMA use in spinal surgery include the lack of a standardized procedure and its liquid nature, which risks diffusion away from the implant site (18).

In 2018, our research group introduced a novel formulation of vBMA—the vBMA clot (19). MSCs derived from clotted vBMA demonstrated superior growth kinetics, increased expression of key growth factors (Transforming Growth Factor Beta, TGFβ; Vascular Endothelial Growth Factor A, VEGF-A; and Fibroblast Growth Factor 2, FGF2), enhanced differentiation potential toward osteogenic and chondrogenic lineages, and reduced expression of Pbx1 and Meis3 genes, which negatively regulate osteoblast development (20). These findings highlighted the superior biological properties of the cellular component within clotted vBMA. The use of clotted vBMA offers distinct advantages over whole and concentrated vBMA, eliminating the need for concentration or purification while providing enhanced stability at the graft site. Given the increasing prevalence of spinal surgery in elderly patients, we also investigated the impact of aging on vBMA clot properties (21). A 2022 study confirmed that donor age does not affect the biological potential of vBMA clot (21). Furthermore, key markers related to aging and cellular senescence were not negatively influenced by age (21). To further validate the clinical application of vBMA clot in SF procedures, a pilot clinical study was conducted at the IRCCS Istituto Ortopedico Rizzoli, involving 10 patients with degenerative spine diseases (Ethical Committee approval n. 587/2020/Sper). At the 12-month FU, the study reported a 100% interbody fusion success rate based on the Brantigan classification and increased bone density from 6 to 12 months postoperatively (13, 22). Patients also experienced significant improvements in the quality of life and health status, as evidenced by clinical scores [Oswestry Disability Index (ODI), Visual Analog Scale (VAS), and EuroQol-5 Level (EQ-5D-5L)] as early as 3 months post-surgery (13).

Despite these promising results, evidence of the true clinical benefits of this treatment strategy remains limited, and no high-level clinical studies have thoroughly investigated its advantages. Furthermore, the preclinical biological, anti-inflammatory, and antibacterial properties of this multifunctional bioscaffold remain underexplored.

A randomized clinical trial (RCT) evaluating the additional benefit of vBMA compared to allogeneic bone alone could provide definitive insights and help establish this combined therapeutic approach as a standard treatment option for patients undergoing SF surgery in the lumbar spine.

### Objectives and trial design

An RCT has been designed to evaluate the efficacy of autologous vBMA clots in SF procedures for patients with lumbar degenerative spine diseases. The study compares two groups: patients treated with autologous vBMA clots combined with bone allograft chips versus those receiving bone allograft chips alone (standard treatment). Additionally, the study assesses whether patient age and gender influence clinical outcomes.

As secondary objectives, this study aims to characterize platelet-specific parameters, cytokines/chemokines, growth factors, pro-inflammatory and anti-inflammatory markers, and MSCs that define the vBMA clot through molecular and cellular analyses. To further investigate its intrinsic biological potential, an advanced humanized three-dimensional (3D) *in vitro* SF model is developed. All experimental setups will consider age and gender differences, including an evaluation of their interactions.

Furthermore, the study examines the antibacterial properties of vBMA clot against common Gram-positive and Gram-negative bacteria associated with spinal infections, including methicillin-sensitive and methicillin-resistant *Staphylococcus aureus* and *Escherichia coli*. *In vitro* time-kill and bacterial adhesion assays are conducted to assess the ability of vBMA clot to inhibit bacterial growth as well as analyze the potential variations related to patient age and gender.

## Methods and analysis

### Study setting

This controlled RCT investigates patients undergoing SF surgery for degenerative spinal diseases (disc disease, lumbar spinal stenosis, grade I and II spondylolisthesis, etc.). The study compares clinical and radiological outcomes between two groups: patients treated with vBMA clot combined with allograft bone chips (Group 1–Experimental) and those treated with allograft bone chips alone (Group 2–Standard). All trial-related activities are conducted at the IRCCS Istituto Ortopedico Rizzoli (Bologna, Italy), a specialized orthopedic center for treating spinal diseases, in compliance with the European Union Clinical Trial Regulation and the Declaration of Helsinki.

### Patient and public involvement

Patient and public involvement (PPI) in clinical trials emphasizes collaboration between researchers and patients, ensuring that studies address patient-centered concerns and priorities. In recent times, PPI extends beyond trials, influencing healthcare resource allocation and research priorities worldwide. Continued and sustainable progress—such as that proposed in the present trial, which utilizes a multifunctional therapeutic strategy based on a safe, functional, and easy-to-use bioscaffold—relies on strong policies, historical awareness, and ongoing evaluation to maintain balance and effectiveness in this partnership.

### Eligibility criteria

The inclusion criteria are men or women aged between 18 and 80 years at the time of surgery and patients with radiologically diagnosed spinal disorders who are candidates for posterior SF involving ≤5 levels, either posterolateral or interbody fusion, with pedicle screw fixation, excluding cases with standalone posterolateral fusion.

The exclusion criteria include patients with systemic infections (e.g., HIV, HBV, or HCV); those with coagulation disorders; pregnant or breastfeeding women; those with oncological diseases; those with local infectious diseases (e.g., tuberculous spondylodiscitis and suppurative spondylodiscitis); a history of previous spinal surgery; those undergoing radiotherapy or chemotherapy; those with myeloproliferative disease; those receiving steroid therapies; those with untreated thyroid disorders; immunosuppressed patients; those with traumatic spinal conditions; and those with osteoporotic conditions affecting spinal integrity.

### Surgical procedures and interventions

During the screening phase, local specialized orthopedic medical staff assess the eligibility of potential participants. Once informed consent is obtained, an external investigator (not involved in the trial) assigns participants to one of the two groups:

- Group 1 (Experimental): vBMA clot combined with allograft bone chips;
- Group 2 (Standard): allograft bone chips alone.

As previously mentioned, the allograft bone chips are supplied by the local Musculoskeletal Tissue Bank, while the vBMA is harvested from the vertebral pedicles during surgical site preparation for pedicle screw insertion. The procedure follows a conventional posterior approach for lumbar SF and utilizes standardized instrumentation. In detail, after pedicle screw placement, decompression of the cauda equina and nerve roots is achieved through hemilaminectomy and foraminotomy, if necessary. Bone tissue removed during site preparation for arthrodesis is considered surgical waste and is not combined with allogeneic bone chips. The assigned treatment—either vBMA clot combined with allograft bone chips or allograft bone chips alone—is applied to the laminae or hemilaminae and the transverse process on the contralateral side of the hemilaminectomy. On the hemilaminectomy side, foramino-arthrectomy is performed to insert the interbody fusion cage, if needed.

**Figure 1:**
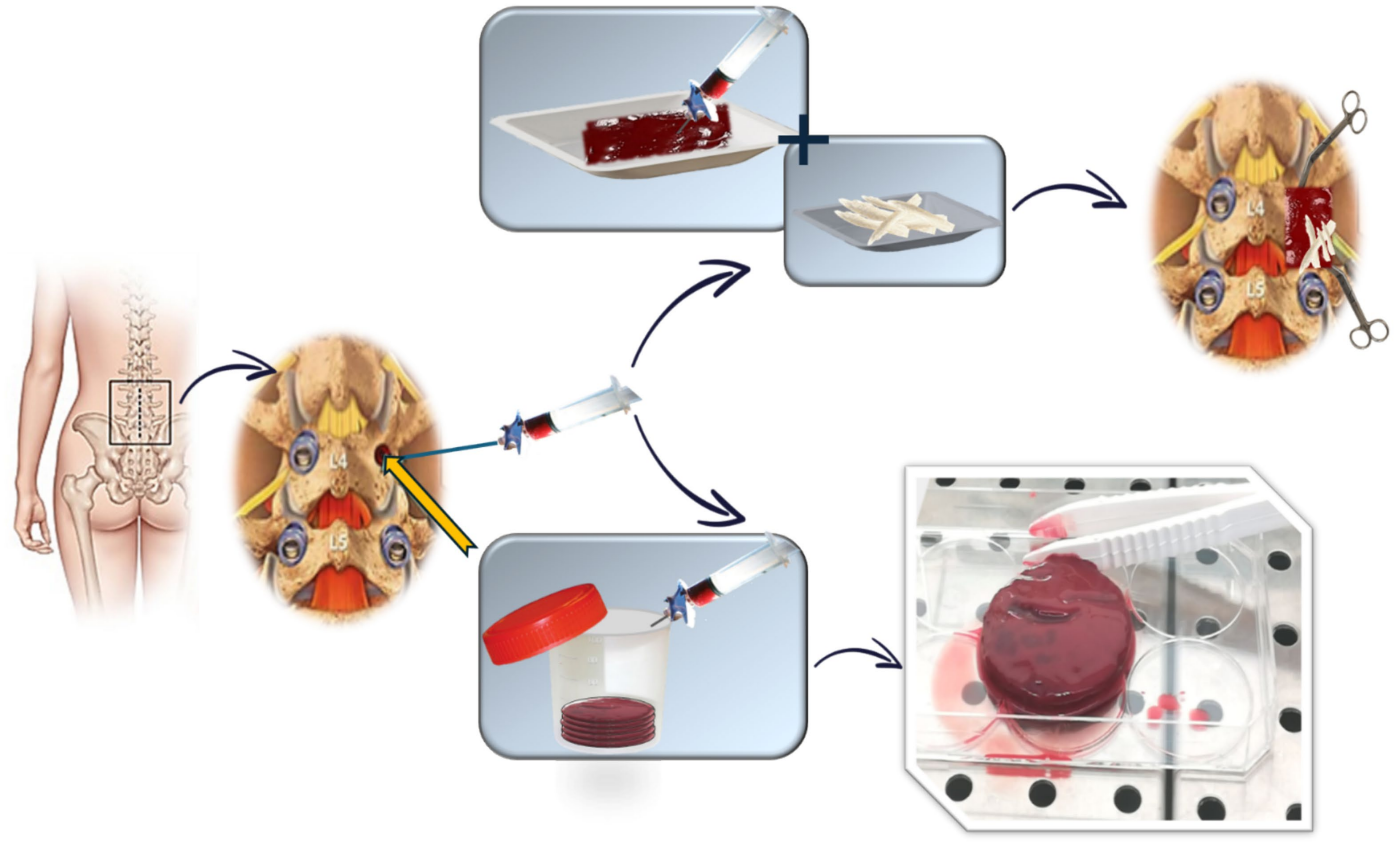
Schematic representation of vBMA combined with allogenic bone chips treatment (experimental group).

**Figure 2:**
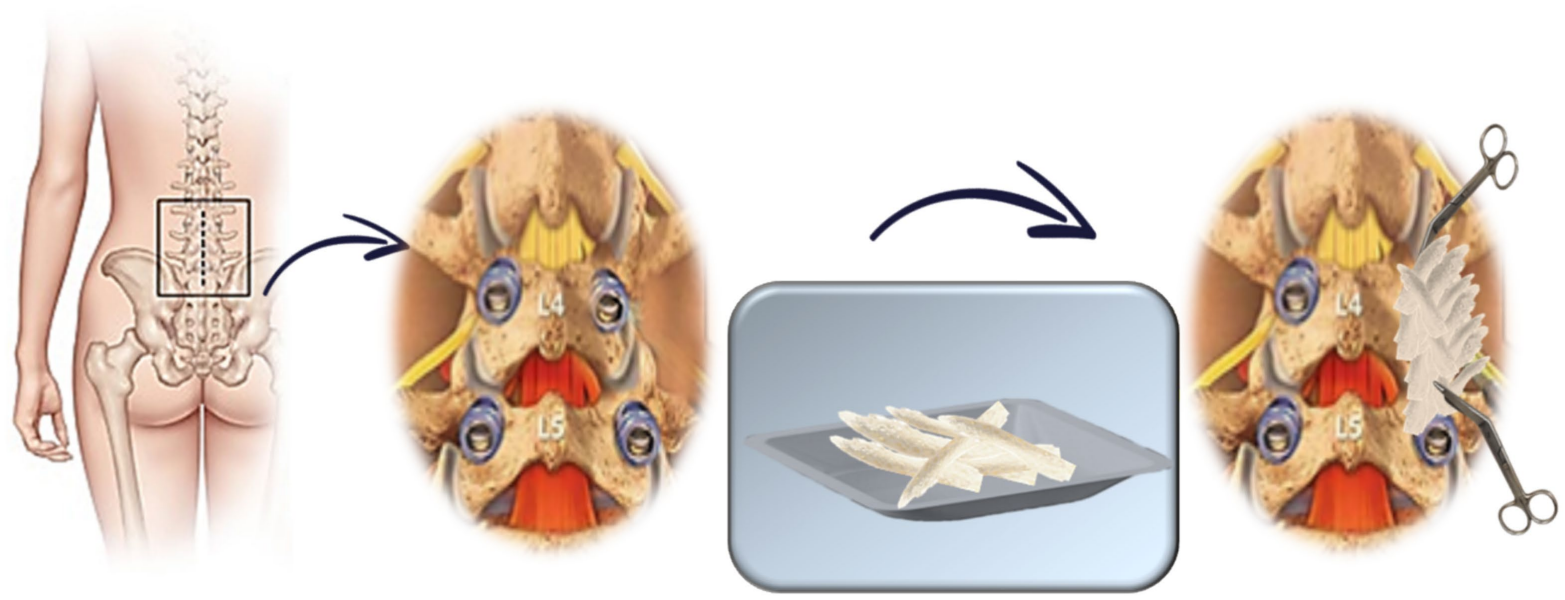
Schematic representation of allogenic bone chip approach (standard group).

Since aspiration volume influences the concentration of progenitor cells in vBMA, a standardized approach is used to maximize the cell yield. A predetermined volume of bone marrow, as defined in the registered patent application, is aspirated from each pedicle involved in the surgery. After aspiration, vBMA is evenly divided into two sterile containers, clotted (clotting time: 15–20 min), and then used for the surgical procedure (Figure 1). For each vertebra, the assigned treatment is administered as follows:

- Experimental group: clotted vBMA combined with 20 g of allograft bone chips per side (Figure 1);
- Standard group: 20 g of allograft bone chips per side (Figure 2).

The total volume depends on the number of spinal segments involved, with a maximum of 200 g of allogeneic bone and a predetermined volume, as defined in the registered patent application, of vBMA for a five-level fusion. Any remaining vBMA is allocated for preclinical *in vitro* studies, as described in the “Outcomes” section (Figure 1).

Postoperative care, including physical therapy and the use of bracing, was not standardized and was left to the discretion of the treating physician according to the patient’s clinical condition and standard hospital practices.

### Outcomes

The primary outcome is to compare the two groups regarding the rate and duration of SF. Radiographic assessments are conducted preoperatively and at 1, 3, 6, and 12 months of FUs. To minimize exposure to ionizing radiation, computed tomography (CT) is performed only at 6 and 12 months, alongside x-rays. A diagnostic and interventional radiologist, blinded to group assignments, evaluates CT scans and x-rays, assigning interbody fusion grade according to the Brantigan classification (ranging from A to E, with E indicating the highest fusion rate) (22). The SF rate is analyzed over the FU period, considering variations due to age and gender differences.

The secondary outcomes include evaluating the pseudoarthrosis rate estimated using the Brantigan classification (22) and clinical outcomes. Moreover, since Brantigan (and other similar) classifications are visual qualitative scales affected by a lack of precise quantification, we provided a new quantitative analysis based on density calculated on CT images. The density of the selected region of interest (ROI) has been calculated and reported in Hounsfield Units (HU) for each study (baseline and follow-up controls). ROIs have been positioned within facet joint spaces of all vertebral levels of interest. Differential values in facet joint density have been reported by comparing baseline with follow-up controls to integrate the Brantigan scale (or help in cases of difficult grading selection), offering a quantitative and reliable tool. Measurements were performed using Picture Archive Communication System (PACS) software version 12.2.6.2000019.

- Clinical outcomes include the following:
- Quality of life improvement was assessed through the VAS (0–10, with higher scores indicating more severe pain), the ODI (0–100, with higher scores indicating greater disability), and the SF-36, a standardized quality of life questionnaire based on patient self-assessment (23–25). These measures are administered at baseline (pre-surgery) and at 1, 3, 6, and 12 months postoperatively. Although patient-reported outcomes such as ODI, VAS, and SF-36 are typically self-reported, a blinded evaluator collected these outcomes to ensure standardization, consistency, and completeness of data across all participants. This approach minimizes potential biases related to symptoms and functioning reporting and enhances the overall reliability of the collected data.
- Postoperative complications are assessed through medical records, with complications classified as early (major and minor) or late based on their time of onset. Re-operation rates and implant removal were also recorded and analyzed as part of the clinical outcomes, indicating surgical complications and long-term treatment success. The results are analyzed for up to 12-month FUs.
- Laboratory parameters include routine blood tests, including red and white blood cell counts, platelet count, mean platelet volume, hemoglobin levels, etc., that are collected preoperatively and postoperatively. Additionally, any comorbidities and adverse events are systematically recorded.

Finally, coagulated vBMA from patients enrolled in the RCT is used to conduct several preclinical *in vitro* studies aimed at further investigating its characteristics, biological potential, and anti-inflammatory and antibacterial properties. These studies also evaluate age and gender differences and include an analysis of their interactions. Specifically, clotted vBMA is employed for the following:

- Developing an advanced 3D *in vitro* model of SF: the vBMA clot is placed between allograft bone chips and cultured *in vitro* to evaluate cell viability, adhesion, distribution, and colonization of the cells within the vBMA clot in the allogeneic bone chips. Biological, molecular, histological, histomorphometric, and immunohistochemical analyses are performed;
- Characterizing, through immunoenzymatic assays and molecular techniques, the expression of platelet-derived growth factors, cytokines/chemokines, and osteotropic and angiogenic factors present in the coagulated vBMA can modulate bone formation, healing, regeneration, vascularization, and inflammation. Additionally, biological and molecular analyses are conducted to characterize the morphology, phenotype, and differentiation potential of MSCs present in the coagulated vBMA;
- Quantifying the antibacterial activity of vBMA can be achieved through microbiological tests using both Gram-positive and Gram-negative bacterial strains, employing microbiological techniques, cell biology methods, and scanning electron microscopy.

### Data collection and management

The necessary clinical data are extracted from the patient’s medical records and source documents, providing essential information on demographics, medical history, diagnosis, treatments, and any adverse event or complications that occurred during hospitalization or FU. A dedicated data collection form is used to systematically record all clinical information and study-required analyses, ensuring consistency, accuracy, and reliability in data gathering. For each enrolled patient, a Case Report Form (CRF) is created. The CRF is a document used in clinical research to collect and manage data in compliance with regulatory requirements and study protocols. It serves as a crucial tool for ensuring data integrity, tracking patient progress, monitoring adherence to the study design, and facilitating statistical analyses. By maintaining a structured approach to data collection, this study aims to enhance the reliability and validity of findings, ultimately contributing to improved patient care and medical advancements.

### Statistical methods

The sample size for the trial was calculated through an *a priori* power analysis using G*Power software (version 3.1.9.6, University of Kiel, Germany), considering a power of 0.80 and a confidence level of 95% (one-tailed Fisher’s exact test). The effect size, in terms of odds ratio, was calculated based on spinal fusion literature data (23, 24). Ricart et al. (26) reported spinal fusion rates of 90% in patients treated with autograft + demineralized bone matrix (DBM), compared to 70% in those treated with allograft and bone marrow aspirate (BMA) (OR = 3.9). Slosar et al. (27) reported SF rates of 93% in patients treated with allograft and bone morphogenetic protein (BMP) compared to 73% of those treated with allograft alone (OR = 5.3). Therefore, the effect size was set at an OR of >4.8, assuming a higher SF percentage in the group treated with vBMA clot combined with allogeneic bone chips (>91%) compared to the group treated with allogeneic bone chips alone (<70%). For each treatment group, the minimum number of patients is *n* = 58, including a 20% drop-out rate.

Statistical analysis is conducted using R software and related packages, such as ggplot2, for graphical data representation. A *p-*value of < 0.05 is considered statistically significant. All data are recorded in an electronic dataset containing continuous variables (e.g., age, growth factor expression, and bacterial time kill results), categorical nominal variables (e.g., gender, diagnosis, and treatment), and categorical ordinal variables (e.g., Brantigan SF classification, VAS score, and ODI score). A univariate analysis should be conducted to determine the distribution, central position (e.g., mean and median), and dispersion (e.g., variance, standard deviation, and interquartile range [IQR]) of quantitative variables. For qualitative variables, frequency distributions are reported. Continuous variables with skewed distributions are transformed and assessed to achieve normality.

To prevent information loss and the introduction of potential selection bias, missing values may be imputed. In cases with very few missing values, a single imputation method is applied; otherwise, a multiple imputation method is applied, providing valid inferences under the missing at random (MAR) assumption. To perform multiple imputations, the Multivariate Imputation by Chained Equations (MICE) R package is used.

After defining and managing the dataset, bivariate or multivariate analyses are carried out to evaluate relationships between variables. In the case of continuous data, the independent Student’s *t*-test and Wilcoxon rank-sum test are used to compare data between the two treatments for parametric and non-parametric data, respectively. Linear models are applied for regression analysis. In the case of categorical dichotomous data, Fisher’s Exact test or chi-squared test is used to compare proportions between the two treatments, and logistic models are applied for regression analysis. For non-dichotomous categorical data, the Mann–Whitney U-test or the Wilcoxon rank-sum test is used to compare ordered outcomes between the two treatments, and the Ordinal Least Squares, Cumulative Link, or Multinomial Logistic models are applied for regression analysis.

Since the variable of the primary outcome is dichotomous (fusion or no fusion), it is analyzed in relation to age (categorized) and gender through logistic regression models. Additional factors (e.g., follow-up time points) and covariates (e.g., platelet-derived growth factors) influencing spinal fusion may be included in the model. To combine covariates into a multiple logistic regression model, collinearity and predictive power are assessed. The results are reported as odds ratios (ORs) with corresponding 95% confidence intervals (CIs). The primary outcome is also assessed through appropriate stratifications of the population based on the presence or absence of specific comorbidities.

Given the longitudinal nature of FU data (e.g., VAS, ODI, and spinal fusion grading across multiple time points), repeated-measures ANOVA or linear mixed-effects models are applied to assess both within-subject (time) and between-group (treatment) effects, accounting for intra-subject correlation and missing data. This finding allows for a robust evaluation of changes over time and treatment efficacy.

Finally, after defining the most reliable models based on multiple criteria (e.g., Akaike Information Criterion, R^2^), diagnostic tools are used to evaluate model assumptions and identify potential influential observations. The same analysis process is implemented for the other regression models related to secondary outcomes, with appropriate diagnostics applied.

Data collection and statistical analysis are standardized and blinded.

### Data monitoring

Effective clinical trial data monitoring is essential for ensuring data integrity, patient safety, and regulatory compliance. By employing rigorous quality control measures, leveraging advanced technologies, and implementing risk-based strategies, researchers can enhance the reliability of clinical trial outcomes while optimizing resource utilization. The monitoring personnel of the data belongs to the local Clinical Trial Center, which is another independent entity of the Institute. An interim report and a final report are planned to be submitted to the Italian Ministry of Health, which funded the project (GR-2021-12372257).

## Ethics and dissemination

### Research ethics approval and consent

Ethical approval was granted on 16 March 2023 by the local Ethics Committee (CE AVEC) (Protocol MORE_FOR_SPINE; Number 149/2023/Sper). All participants will provide informed written consent during their baseline outpatient medical examination with trained medical staff, before enrollment. Participation in the trial is entirely voluntary, and participants may withdraw at any time.

### Protocol amendments

Minor protocol amendments, such as database modifications to enhance monitoring processes or improve outcome assessment through questionnaires, will be fully documented. Major amendments, including changes to the patient information sheet or consent form, the replacement of a local project leader, or the addition of a new project site, will be submitted to the Ethics Committee for approval.

### Confidentiality and access to data

In accordance with the General Data Protection Regulation (GDPR) (EU Regulation 2016/679) and Clinical Trial Regulation 536/2014, data in this study are recorded, processed, stored, and managed while ensuring confidentiality through appropriate technical and organizational measures.

Patients involved in the project are not identified by name or any other personal information in study reports, documents, or files. Instead, only a unique code number is recorded. A separate file linking each patient’s code to their personal data (e.g., name, clinical chart number, telephone number, and address) is securely stored within the local Information System. This file, along with all static (uneditable) and dynamic (editable) RCT and preclinical files, data, records, and images/microfilms, is kept in a password-protected archive folder.

The archive is then saved on a cloud server managed by the local institute data protection officer, with access granted through a one-step login and password at different user-access levels. Only key project personnel can access individual data files. Additionally, a backup copy of the archive is created and stored every 2 weeks by the data protection officer.

For data available only in paper form (or other non-electronic media), both in RCT and preclinical studies, documents are scanned, converted into static PDF format, and stored following the same procedures as electronic files. The original paper documents are securely archived for 10 years after the project’s completion, under the supervision of the research team.

### Dissemination policy

This trial has been designed and is being conducted in accordance with international standards of Standard Protocol Items: Recommendations for Interventional Trials (SPIRIT), which guide the development of clinical trial protocols before study initiation. Once the results are available, they will be reported following the Consolidated Standards of Reporting Trials (CONSORT) guidelines to ensure transparent and comprehensive dissemination. The results will be shared through peer-reviewed publications and presented at national and international conferences. Authorship will follow the 2018 recommendations of the International Committee of Medical Journal Editors.

### Scientific relevance and broader impact

The main challenge of SF surgery is to have a safe, functional, high-quality, and easy-to-use therapeutic strategy to improve surgery success and health-related quality of life, as well as in those patients with higher revision rates, such as female and elderly patients. The biological steps involved in creating a bony fusion between adjacent segments of the spine are a complex and highly coordinated series of events. In this context, the relevance of this research is to provide evidence on the potentiality of the vBMA clot as an autologous and multifunctional bioscaffold that can target all the main key challenges for SF surgery. First, the vBMA clot may act as an osteogenic and osteoinductive 3D bioscaffold containing pluripotent mesenchymal and hematopoietic stem cell that work synergistically to foster bone formation and regeneration. Second, platelet degranulation in the vBMA clot allows the release of various biomolecules (alpha granules, platelet-specific proteins, cytokines/chemokines, growth factors, coagulation factors, and adhesion molecules), which may promote early vascularization, a process vital for bone homeostasis, healing, regeneration, and the osseointegration of fixation hardware. Third, mesenchymal progenitors in the vBMA clot may modulate inflammation through a paracrine immunomodulatory effect, enabling an optimal transient stage of acute inflammation, which is a key element for successful bone healing. Fourth, an additional powerful element of the vBMA clot could be the critical role of the coagulation cascade and bone marrow MSCs in the early innate immune system activation and in their involvement in stressing and eliminating bacteria. The ability of the vBMA clot to provide a local combined delivery system of stem cells, signaling biomolecules, and anti-inflammatory and antibacterial factors, all enclosed by a matrix molded by the clot, represents an advanced and simple strategy to meet the main clinical needs of SF. vBMA is harvested simultaneously during the preparation of the site for pedicle screw insertion, and after clotting (~15–20 min), it is reintroduced in association with a bone allograft at the fusion site, without additional surgical time or donor site involvement. This approach offers a single, safe treatment while eliminating the need for *ex-vivo* cell manipulation, synthetic induction factors, or complex fabrication processes, thus providing a ‘One-Health’ strategy for SF surgery with a significant impact on patients.

## Potential limitations

This study protocol may have several potential limitations. Although a 12-month follow-up period is generally sufficient to assess early fusion and clinical outcomes, a longer follow-up, ideally 24 months, would allow for a more comprehensive evaluation of long-term fusion stability and the durability of clinical benefits. The monocentric design could introduce center-specific biases related to surgical technique and perioperative management, potentially affecting the generalizability of the results. Additionally, while age and sex are considered, other relevant confounding factors, such as comorbidities, smoking status, bone mineral density, and concurrent pharmacological treatments, may not be fully controlled and could influence both radiological and clinical outcomes. Despite standardized imaging protocols, radiological evaluation may still carry a degree of interpretative variability. Furthermore, the heterogeneity of the patient population in terms of baseline pathology and surgical indications may introduce variability in the outcomes. Finally, although the biological composition of the vBMA clot is analyzed, its correlation with individual patient outcomes is not currently planned, which may limit insights into inter-patient variability in biological efficacy.

## Glossary

SF: Spinal Fusion
BMA: Bone marrow aspirate
vBMA: Vertebral Bone Marrow Aspirate
RCT: Randomized Clinical Trial
FU: Follow-Up
CT: Computed Tomography
VAS: Visual Analog Scale
ODI: Oswestry Disability Index
SF-36: Short Form 36 Health Survey
MSCs: Mesenchymal Stem Cells
BGLAP: Bone Gamma-Carboxyglutamate Protein
ALPL: Alkaline Phosphatase
RUNX2: Runt-Related Transcription Factor 2
TGFβ: Transforming Growth Factor Beta
VEGF-A: Vascular Endothelial Growth Factor A
FGF2: Fibroblast Growth Factor 2
3D: Three-dimensional
PPI: Patient and public involvement
HIV: Human Immunodeficiency Virus
HBV: Hepatitis B Virus
HCV: Hepatitis C Virus
ROI: Region of interest
HU: Hounsfield Unit
PACS: Picture Archive Communication System
CRF: Case Report Form
BMP: Bone morphogenetic protein
IQR: Interquartile Range
MICE: Multivariate Imputation by Chained Equations
OR: Odds ratio
CI: Confidence intervals
CE: Ethics Committee
GDPR: General Data Protection Regulation
